# The future … to whom it belongs?^☆^

**DOI:** 10.1016/j.bjorl.2016.05.001

**Published:** 2016-05-16

**Authors:** Marcio Abrahão

**Affiliations:** Department of Otorhinolaryngology and Head and Neck Surgery, Escola Paulista de Medicina, Universidade Federal de São Paulo (UNIFESP), São Paulo, SP, Brazil

The aim of an editorial is to make us think! I chose, from among several topics, one that I consider very important for us to reflect on together: being a physician today.

We work in our profession, intensely, for many hours of the day. Treating public or private patients, while others are doing research, teaching and even working with institutional management.

As the majority works caring for patients, I will prioritize this discussion.

Patient care in public institutions is in a chaotic state. Medical schools are being created throughout Brazil, without at least having a teaching hospital, which is our true classroom. School–hospitals that coexist with other public hospitals are in a state of dereliction, regarding equipment, without medications for a reasonable care to be offered, in contrast to a demand that grows exponentially.

We face endless lines and shifts with poor conditions of service.

What about patients from private health plans and health insurance?

To answer this question, I remember how many children with adenotonsillectomy indication due to upper airway obstruction and even apnea are not operated due to a low medical honorarium. While patients with health insurance undergo procedures with the abusive use of laser, navigators, robotic surgery, nerve monitoring, biological glues, sophisticated tools and products for hemostasis. All these increase costs unnecessarily, leading to an “arm wrestling” between private institutions, doctors and health insurance companies.

We are not against the advances of technology in favor of our patients, but the sustainability of supplementary health depends on good management.

We cannot forget that this entire context is properly seasoned with the judicialization of our service. There is an actual increase in the number of lawsuits against physicians and legal interventions for this or that patient to be treated or operated on.

We cannot forget the huge taxes that are imposed on us.

As for those working with research, the decrease in funds from research development institutions has been enormous, preventing high-level research to be carried out and stopping many that had already started.

The physicians who dedicate their lives to teaching never had such low wages and inadequate working conditions.

Well, many will say about this catastrophe that the future belongs to God! A popular proverb very commonly heard.

I do not think so. The future belongs to us, doctors, with energetic and precise attitudes. The cohesion of the team is essential. Within our specialty, this can be achieved with the joining of all otorhinolaryngologists to the ABORL-CCF, which is our official representative organ.

Increasing the number of workers’ cooperatives in small towns, changing the current fees, ensuring the quality of medical education and specialties… We have to appreciate the effort we have made to become doctors and how much we love and are dedicated to our profession.

Politics is not made exclusively in Brasilia; it is made mainly in the communities, in institutions. We have to maintain our ethics while being respected, by occupying spaces. If we do not occupy them, others will do it.

## Conflicts of interest

The author declares no conflicts of interest.

